# Disability and Fatigue Can Be Objectively Measured in Multiple Sclerosis

**DOI:** 10.1371/journal.pone.0148997

**Published:** 2016-02-10

**Authors:** Caterina Motta, Eduardo Palermo, Valeria Studer, Marco Germanotta, Giorgio Germani, Diego Centonze, Paolo Cappa, Silvia Rossi, Stefano Rossi

**Affiliations:** 1 Dipartimento di Medicina dei Sistemi, Tor Vergata University, Rome, Italy; 2 IRCCS Istituto Neurologico Mediterraneo (INM) Neuromed, Pozzilli, Italy; 3 Department of Mechanical and Aerospace Engineering, “Sapienza” University of Rome, Italy; 4 Fondazione Santa Lucia/Centro Europeo per la Ricerca sul Cervello (CERC), Rome, Italy; 5 Don Carlo Gnocchi Onlus Foundation, Milan, Italy; 6 Neuroimmunology and Neuromuscolar Diseases Unit, IRCCS Foundation Carlo Besta Neurological Institute, Milan, Italy; 7 Department of Economics and Management–Industrial Engineering, University of Tuscia, Viterbo, Italy; University of Oxford, UNITED KINGDOM

## Abstract

**Background:**

The available clinical outcome measures of disability in multiple sclerosis are not adequately responsive or sensitive.

**Objective:**

To investigate the feasibility of inertial sensor-based gait analysis in multiple sclerosis.

**Methods:**

A cross-sectional study of 80 multiple sclerosis patients and 50 healthy controls was performed. Lower-limb kinematics was evaluated by using a commercially available magnetic inertial measurement unit system. Mean and standard deviation of range of motion (mROM, sROM) for each joint of lower limbs were calculated in one minute walking test. A motor performance index (E) defined as the sum of sROMs was proposed.

**Results:**

We established two novel observer-independent measures of disability. Hip mROM was extremely sensitive in measuring lower limb motor impairment, being correlated with muscle strength and also altered in patients without clinically detectable disability. On the other hand, E index discriminated patients according to disability, being altered only in patients with moderate and severe disability, regardless of walking speed. It was strongly correlated with fatigue and patient-perceived health status.

**Conclusions:**

Inertial sensor-based gait analysis is feasible and can detect clinical and subclinical disability in multiple sclerosis.

## Introduction

Disability assessment had become fundamental part of standard multiple sclerosis (MS) practice and clinical research. Selecting an appropriate outcome measure for clinical trials is important in determining whether the intervention is actually modifying the disease course, especially because in MS the concept of relapse-free disease has been shifting into disease-free status, thus requiring more sensitive metrics to determine efficacy. Moreover outcome measures and surrogate endpoints have been applied to the individual patient to evaluate disease progression and the need for a change in therapy.

While disability may be considered an ideal primary endpoint in clinical research and a therapeutic target in clinical practice, it can be difficult to both define and measure. The available clinical measures of disability in MS, including the expanded disability status scale (EDSS) [1] and the MS Functional Composite (MSFC) [2] are not responsive and sensitive [3],[4]. Inter-rater variation has been reported to be greater than a 1-point increase in the EDSS about 40% of the time and difficulty in quantifying a meaningful change in the MSFC components has been claimed [3]. An additional challenge for determining clinical meaningfulness is the dichotomy between the patient's and the clinician's perceptions of change and the significant impact of fatigue in the limitation of performance. Among MS patients, fatigue is the most commonly reported symptom and one of the most debilitating, with significant socioeconomic consequences [5], but it is often under-emphasized because of its complexity and subjective nature. The International Advisory Committee on Clinical Trials in MS has recently pointed out the need for novel collaborative approach to most effectively measure disability, including the use of composite endpoints and patient-reported outcomes [4].

In literature, several studies were focused on the assessment of motor disabilities in patients with MS by means of stereophotogrammetric systems [6–8]. However, these systems require closed and restrained laboratories, they are expensive in terms of time and finance and they are not useful in studies involving a large group of subjects [9]. In order to overcome the reported drawbacks, Magnetic Inertial Measurement Units (MIMU/IMU) can be used instead of stereophotogrammetric systems. In fact, they can be utilized in wider and outdoor workspaces, they do not require long lasting procedures for their use, and they are low cost devices [10]. In a recent review on the use of MIMUs to objectively quantify motor disabilities of subjects with neurological diseases, only one study was focused on subjects with MS [11]. In particular, the authors found differences between subjects with MS and controls during gait, analyzing only the range of motions of trunk angles. To the best of our knowledge, no studies have been conducted in order to provide objective outcome measure for the evaluation of lower limb motor disabilities in subjects with MS by means of MIMUs.

Aim of this study was to investigate the feasibility of gait analysis in MS, by using commercial wearable inertial sensors, and to establish novel and sensitive observer-independent measures of disability.

## Methods

This study complied with the principles of the Declaration of Helsinki, and was approved by the Ethical Committee of the Policlinico Università Tor Vergata in Rome. All the subjects gave their written informed consent to the study.

### Subjects and Study Procedures

A total of 130 subjects was included in this study. Eighty patients with a diagnosis of MS [12], were recruited by the MS Center of the Tor Vergata University Hospital of Rome. Fifty-six had relapsing-remitting MS (RRMS), and 24 a secondary progressive MS (SPMS) [13]. Patients could not be enrolled if they relapsed in the 60 days preceding inclusion. Patients with EDSS>6.5 or unable to complete the walking trial without aid were excluded. Other exclusion criteria were: the need for an orthosis for stance control of the foot, ankle, and/or knee, the receipt of botulinum toxin injections in the lower extremity within the preceding 6 months, the use of a baclofen pump with unstable dosing in the last 3 months, a diagnosis of peripheral nerve injury in the involved lower extremity with symptoms that limited participation in study activities, or receipt of dalfampridine for the treatment of MS symptoms. Fifty age and sex-matched subjects without neurological or other relevant medical conditions served as a reference population. Demographic and clinical details were derived from medical records and shown in Table 1.

**Table 1:** Demographic and Clinical Characteristics of Subjects.

	Total	HC	MS	RRMS	SPMS
Number	130	50	80	56	24
Age (years)	33.9±10.4	33.0±10.7	34.5±10.3	30.0±8.2	45.1±6.0
Sex (M/F)	53/77	20/30	33/47	21/35	12/12
Disease duration (years)	-	-	8.1±6.6	4.9±2.9	15.7±6.6
EDSS	-	-	3.3±1.6	2.5±1.3	5.1±0.8

M: male; F: Female; HC: Healthy Control; MS: Multiple Sclerosis; RRMS: Relapsing Remitting Multiple Sclerosis; SPMS: Secondary Progressive Multiple Scleorosis; EDSS: Expanded Disability Status Scale.

MS disease onset was defined as the first episode of focal neurological dysfunction indicative of MS. Disease duration was estimated as the number of years from onset to the inclusion.

Patients underwent examination comprehensive of clinical assessment of disability, gait analysis, questionnaires within 24 hours with at least 1 hour between the assessments. Healthy controls (HC) only underwent gait analysis. Gait analysis was performed twice, a day apart, to assess retest reliability.

### Clinical Disability Assessment

Disability was determined by a specially trained and certified examining neurologist using Expanded Disability Status Scale (EDSS) [1]. The timed 25 Foot Walk (T25FW), which measures the time a patient requires to cover a distance of 25 feet at maximum speed, was also recorded. Clinical assessment of fatigue was performed by the same examining neurologist, unaware of kinematic results, to cathegorize patients into two groups (patients with fatigue versus patients without fatigue) to assess the ability of gait analysis to discriminate patients with fatigue respect to questionnaire score. Motor fatigue was assumed if the patient reported an abnormal rapid physical exhaustion in daily living and if a severe reduction in maximum gait distance could not be explained by the degree of paresis, spasticity or ataxia [14].

### Gait Analysis

**Fig 1:**
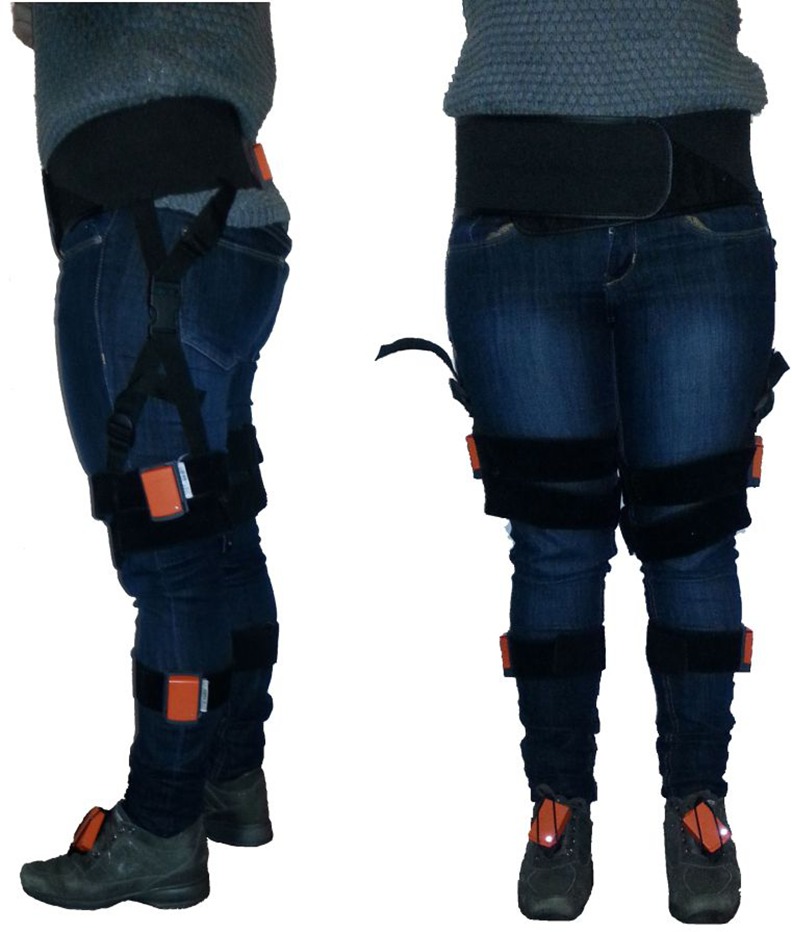
Position of each MIMU on one subject.

Participants were instructed to walk at a comfortable and self-selected speed along a leveled walkway of 15 m for one minute. We defined the start and the end of the gait path with two turn lines where the subjects reversed their walking direction by 180°. Walking trial was repeated three times and the mean of kinematic variables along the trials were analyzed. Subject that experienced fatigue at the end of each walking trial were allowed to rest on a chair until they felt ready to perform the next repetition. Subjects were equipped with seven wireless MIMUs (Xsens MTw, Xsens Technologies, Enschede, The Netherlands) placed on pelvis and on thigh, shank and foot of both legs (Fig 1). The effects of magnetic field distortions on MIMU outputs were neglected moving any movable ferromagnetic materials out from the experiment area [15]. The relative orientations of each MIMU and the related body segment have been evaluated by means of a validated functional calibration procedure [16]. Specifically, it consisted in the gathering of the sensor outputs for five seconds while the subject was keeping still in two different positions: a standing upright posture and a sitting position with the trunk backwards inclined and the legs stretched.

Data processing and data analysis were performed using MATLAB software (MathWorks, USA). The acquired data from MIMUs were post-processed in order to assess the angles of hip, knee and ankle for both legs. Trials were then partitioned in gait cycles. Data related to turning phases and to two strides after and before each turn were excluded from the evaluation, because the lower limb kinematics could be altered by the turning event. Angle curves related to each stride were resampled and normalized at 100 samples. Range of motion (ROM) of joint angle curves on the sagittal plane was computed for each stride.

For each gait trial and each leg, means and standard deviations of ROM of hip (mROM_H_, sROM_H_), knee (mROM_K_, sROM_K_) and ankle (mROM_A_, sROM_A_) were evaluated. As index of the overall gait variability, the E index was calculated as sum of all sROMs related to each joint of both legs. Higher is E value and higher is the variability of gait.

To estimate the asymmetry of kinematics of each i-th joint, we evaluated the symmetry indices SI_H_, SI_K_ and SI_A_ defined as [17]:

SIi=|mROMi-R−mROMi−L|12(mROMi-R+mROMi−L)⋅100

Where right and left limb have been addressed as R and L, respectively. Finally, in order to evaluate the overall gait asymmetry we also calculated:

$$\text{SI}={\text{SI}}_{\text{H}}+{\text{SI}}_{\text{K}}+{\text{SI}}_{\text{A}}$$

The SI value represents the magnitude of asymmetry between legs during the gait. The SI value ranges from 0 to 600% and higher values represent a greater difference between the two sides.

The parameters mROM_H_, mROM_K_, mROM_A_, SI and E were addressed as kinematic variables.

### Questionnaires

Fatigue, quality of life and patient-perceived gait impairment were assessed by validated questionnaires.

Fatigue was assessed by the Modified Fatigue Impact Scale (MFIS) [18] and the Fatigue Severity Scale (FSS) [19] with higher scores indicating grater impact of fatigue on patient functions and daily life. The Patient Reported Indices for MS (PRIMUS) consists of three independent scales; symptoms, activity limitations and QoL designed to be used as standalone measures or in combination. For the present study data were available for the QoL and activity limitation scales. Both scales have been shown to be unidimensional and to have good reproducibility and validity [20].

The impact of MS on the participants' perceived walking ability was assessed using the 12-item Multiple Sclerosis Walking Scale (MSWS-12) [21]. The MSWS-12 provides a score with larger values indicating a greater perception of walking difficulty.

### Statistical Analysis

Test-retest reliability was analyzed using intra-class correlation coefficients (ICC) with an ICC(2,3) model. The between session reliability was analyzed using the mean parameters calculated for the three walking trials for each session. The reliability was classified as excellent (ICC ≥ 0.90), very good (ICC ≥ 0.80), good (ICC ≥ 0.70), moderate (ICC ≥ 0.6) or poor otherwise. Continuous variables are summarized as mean ± standard deviation (SD). Categorical variables are expressed as percentages. To determine differences between two groups, Student's t-test or Mann-Whitney U test was used for continuous variables and the Fisher Exact test was used for categorical variables. Analysis of variance (ANOVA) model was used to compare across groups with different EDSS values. Univariable associations between kinematic variables and clinical disability scales were investigated using Pearson’s correlation analysis or Spearman’s Rho analysis, as appropriate. Kinematic variables with significant association in univariable analysis were used to construct multivariable models, thus including also other measurements of disability and possible confounding factors as age, gender, disease duration, disease course. A ROC curve analysis and the area under the curve (AUC) were used to assess the discriminating ability of the model. An area of 100% represents a perfect discrimination, while an area of 50% represents a worthless model.

A p<0.05 was deemed significant. All tests should be understood as exploratory data analysis as no prior power calculation and subsequent corrections for multiple testing were applied.

## Results

### Reproducibility of Lower Limb Kinematics

Gait analysis was performed twice, a day apart, to assess the retest reliability. Lower limb kinematics showed excellent retest reliability in HC and MS, as reported in Table 2.

**Table 2:** Intra-class Correlation Coefficients of Lower Limb Kinematics.

Kinematics	HC	MS
mROM_H_	0.983	0.976
mROM_K_	0.985	0.997
mROM_A_	0.995	0.997
SI	0.969	0.984
E	0.825	0.877

HC: Healthy Control; MS: Multiple Sclerosis; mROM: mean Range Of Motion; SI: Symmetry Index.

### Analysis of Lower Limb Kinematics in MS Patients

**Fig 2:**
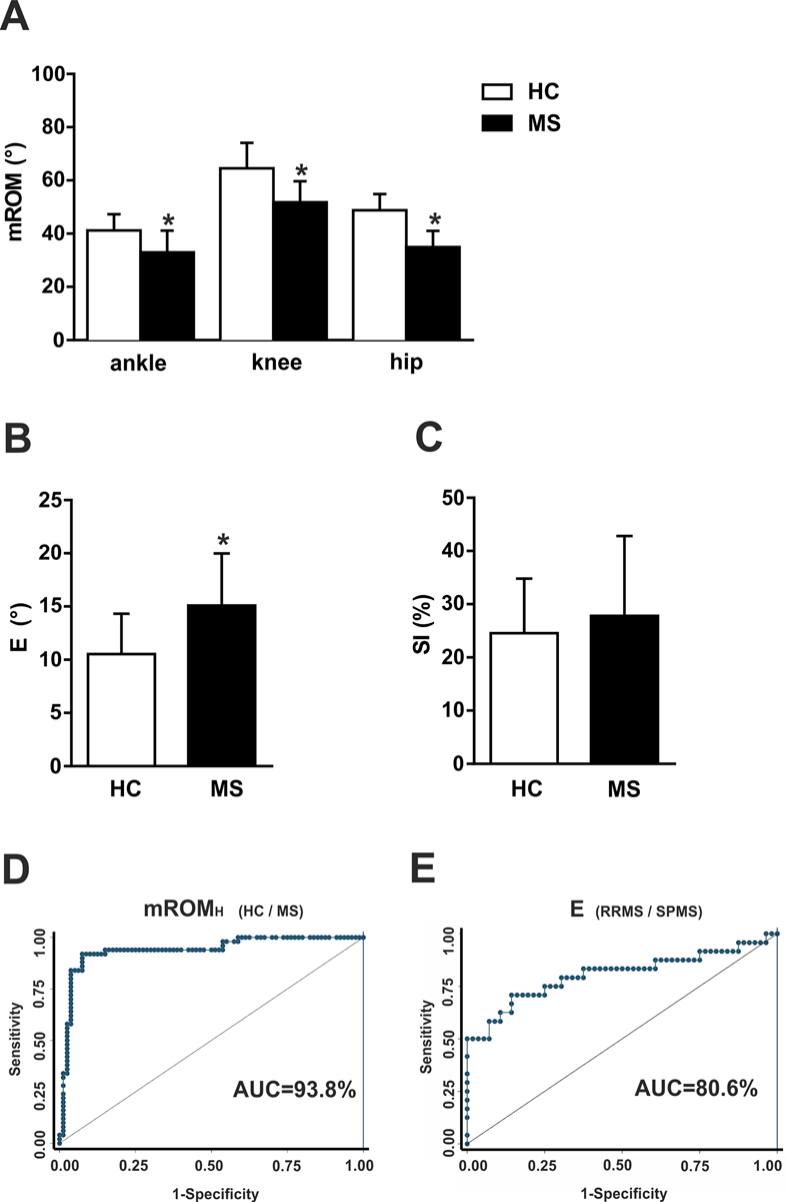
Lower limb kinematics alterations in MS patients. Lower limb kinematics alterations in MS patients. (A) The graph shows thatmROM was significantly lower in MS group respect to HC group in each joint. (B) MS patients presented significantly higher variability of gait, as documented by E analysis. (C) SI was not significantly different between MS and HC group. (D), (E) Area under the ROC curves measured the accuracy of mROMH in discriminating HC from MS (D) and the accuracy of E index in discriminating RRMS from SPMS (E). * means p<0.05.

Five gait parameters were derived from the lower limb kinematics analysis and compared between HC and MS patients. Reduced mROM_H_, mROM_K_, and mROM_A_, were found in MS group with respect to HC group (p<0.05 for each comparisons; Fig 2A). MS patients presented also a significantly higher variability of gait, as documented by E index (p<0.05; Fig 2B). Conversely, SI was not significantly different between the two groups, although higher among MS subjects (p>0.05; Fig 2C).

We then included all the gait parameters significantly different between the HC and MS group in a multivariable model with group as dependent variable, and demographics as possible confounding factors. Two parameters, mROM_H_ and E were found to be independently able to discriminate the MS from the HC group (Table 3). The discriminating ability of mROM_H_ was higher, as assessed by the ROC analysis (mROM_H_: AUC = 93.8%, 95%CI = 89.2–98.5%, Fig 2D; E: AUC = 79.2%, 95%CI = 71.0–87.3%).

**Table 3:** Multivariable Models: Association Between Kinematic Parameters and MS Disease.

MS/HC	OR	SE	p	CI
Age	0.94	0.03	0.09	0.89–1.01
Sex	2.14	1.5	0.28	0.54–8.52
mROM_A_	0.92	0.04	0.07	0.83–1.01
mROM_K_	0.97	0.03	0.53	0.91–1.05
mROM_H_	0.75	0.05	<0.0001	0.65–0.87
E	1.17	0.09	0.038	1.01–1.37
**SPMS/RRMS**				
Age	1.49	2.10	0.005	1.12–1.98
Sex	0.93	1.26	0.96	0.06–13.27
mROM_A_	0.84	0.09	0.11	0.67–1.04
mROM_K_	0.85	0.09	0.12	0.69–1.04
mROM_H_	0.79	0.17	0.27	0.52–1.20
E	1.43	0.22	0.02	1.06–1.93

MS: Multiple Sclerosis; HC: Healthy Control; SPMS: Secondary Progressive Multiple Scleorosis; RRMS: Relapsing Remitting Multiple Sclerosis; OR: odds ratio; SE: standard error; CI: confidence interval; mROM: mean Range Of Motion.

Finally, gait parameters were compared between RRMS and SPMS patients. Gait variability was significantly higher in progressive patients (E: 18.9±5.0° versus 13.4±3.8°; p<0.05), whereas mROMs were significantly lower (mROM_H:_ 31.0±3.0° versus 36.6±6.3°; mROM_K_: 44.4±6.9° versus 54.9±6.0°; mROM_A_: 27.2±7.0° versus 35.4±7.4°; p<0.05). No difference was revealed in SI analysis (RRMS: 27.1±16.5%, SPMS: 29.4±11.0%; p>0.05). All kinematic parameters significantly different between RRMS and SPMS group were included in a multivariable model with groups as dependent variable, and demographics as possible confounding factors. E was the only independent variable associated to progressive disease in our sample, as showed in Table 3, with a high discriminating ability assessed by the ROC analysis, even higher than the standardized measure of motor impairment (E AUC = 0.81, 95%CI = 0.68–0.93, Fig 2E; T25FW AUC = 0.74, 95%CI = 0.61–0.87).

### Correlation between Hip mROM and Disability

**Fig 3:**
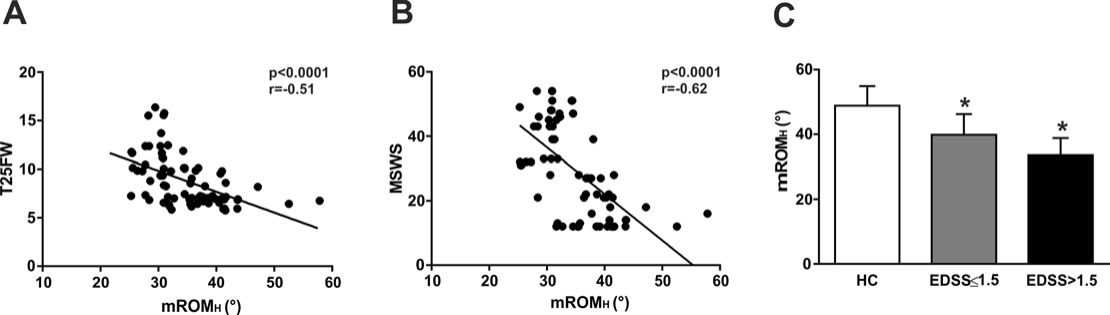
mROM_H_ detects subclinical motor disability. (A) mROM_H_ significantly correlated with T25FW. (B) Significant correlation was found between mROM_H_ and patient-perceived gait impairment assessed by the MSWS questionnaire. (C) mROM_H_ was significantly altered in MS subjects with clinical disability (EDSS>1.5) and without clinical disability (EDSS≤1.5) compared to HC subjects. * means p<0.05.

The correlations of mROM_H_ with clinical variables are shown in Table 4. In particular, mROM_H_ significantly correlated with pyramidal subscore and with T25FW (p<0.05; Fig 3A), as a measure of lower limb motor impairment. Multivariable models confirmed the association between mROM_H_ and EDSS, including demographics and clinical characteristics as possible confounding factors (Coef = -0.08, 95%CI -0.01–0.04, p<0.01) but failed to confirm significantly association at equal value of pyramidal score and T25FW (Coef = 0.01; p>0.1), indicating mROM_H_ as index of motor impairment rather than an index of overall disability.

**Table 4:** Lower Limb Kinematics Correlation with Clinical and Patient-reported Outcomes.

Questionnaire/Scale	mROM_H_		E	
	r	p	r	p
FSS	-0.13	0.23	0.60	<0.001
MFIS	-0.11	0.30	0.63	<0.001
MFIS_physical_	-0.07	0.55	0.67	<0.001
MFIS_cognitive_	-0.12	0.28	0.48	<0.001
MFIS_psycosocial_	-0.14	0.21	0.63	<0.001
MSWS	-0.62	<0.001	0.46	<0.001
PRIMUS_QoL_	-0.17	0.12	0.72	<0.001
PRIMUS_ActivityLimitation_	-0.11	0.33	0.63	<0.001
EDSS	-0.59	<0.001	0.58	<0.001
Pyramidal subscore	-0.61	<0.001	0.57	<0.001
T25FW	-0.51	<0.001	0.10	0.36

mROM_H_: mean range of motion of hip; FSS: Fatigue Severity Scale; MFIS: Modified Fatigue Impact Scale; MSWS: Multiple Sclerosis Walking Scale; PRIMUS: Patient Reported Indices for Multiple Sclerosis; EDSS: Expanded Disability Status Scale; T25FW: Timed 25 Foot Walk.

Further, significant correlation was found between mROM_H_ and patient-perceived gait impairment assessed by the MSWS questionnaire (p<0.05; Fig 3B). In line with previous data showing high sensitivity in detecting MS status, we analyzed mROM_H_ among subjects dichotomized as asymptomatic (EDSS 0–1.5) or symptomatic (EDSS>1.5). Interestingly, mROM_H_ was significantly altered also in MS subjects without clinical disability (p<0.05; Fig 3C).

### Correlation between E Index and Disability

**Fig 4:**
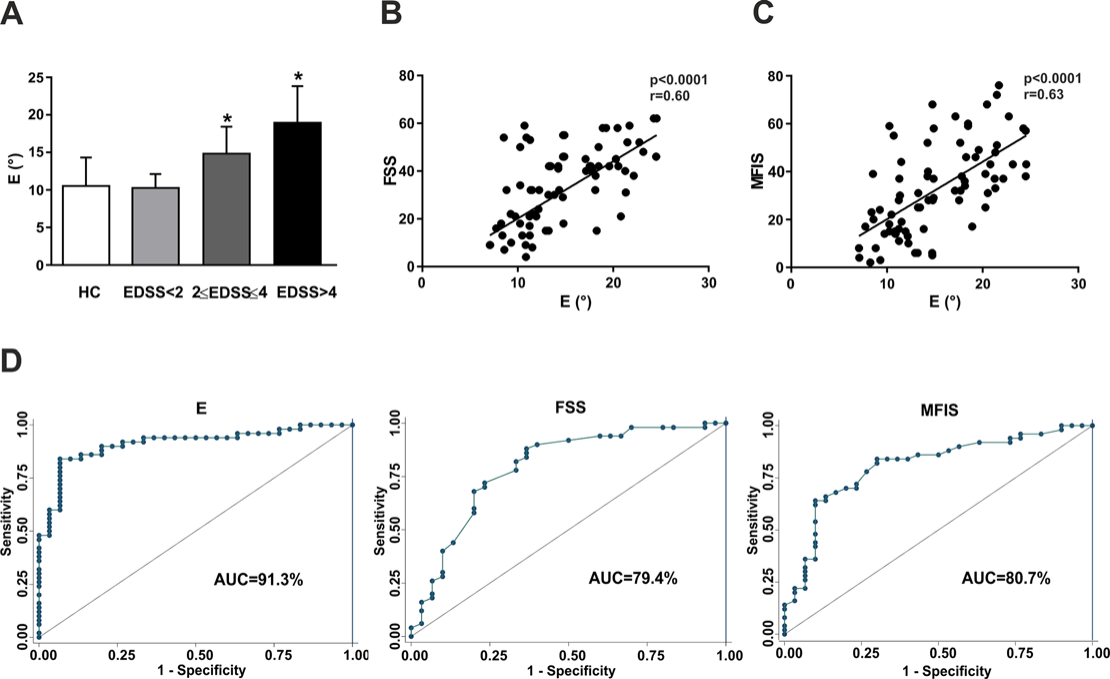
E index discriminates MS patients according to disability and fatigue. (A) A significantly increased of E was found passing from asymptomatic patients (EDSS<2), to patients with EDSS = 2.0–4.0 and patients with EDSS>4.0. (B), (C) E index significantly correlated with fatigue assessed by both FSS (B) and MFIS (C). (D) Area under the ROC curves measured the accuracy of E index, FSS and MFIS in discriminating patients according to the presence of fatigue. * means p<0.05.

We then analyzed gait variability according to different levels of disability. An increased of E was found with increasing disability, passing from asymptomatic patients, to patients with EDSS = 2.0–4.0 and patients with EDSS>4.0 (p<0.05; Fig 4A). Of note, no difference was evident between HC and asymptomatic MS patients (p>0.05). A multivariable model confirmed the association between E and EDSS, at equal value of disease duration, age, sex, disease course (Coef = 0.074, 95%CI = 0.01–0.13, p = 0.017), indicating a correlation with disability also in RRMS patients.

E index significantly correlated with EDSS and pyramidal score, but not with T25FW, as shown in Table 4. A multivariable model confirmed the association between E and EDSS, at equal value of pyramidal score (Coef = 0.064, 95%CI = 0.03–0.09, p = 0.02), proposing E as index of overall disability, also regardless motor impairment.

Furthermore, significant correlations were found with fatigue assessed by both FSS and MFIS (p<0.05, Fig 4A and 4C). A multivariable model confirmed the association between E and the clinical assessment of fatigue (OR = 1.59, SE:0.19, p<0.0001, 95%CI = 1.27–2.02), including demographics and clinical variable (EDSS and pyramidal subscore) as possible confounding factors. The ability to discriminate patients with fatigue was revealed by ROC analysis and it was even higher respect to questionnaire scores (AUC_E_ = 0.91, 95%CI_E_ = 0.84–0.97; AUC_FSS_ = 0.79, 95%CI_FSS_ = 0.68–0.90; AUC_MFIS_ = 0.81, 95%CI_MFIS_ = 0.70–0.91; Fig 4D).

Finally, E correlated with quality of life, activity limitation and perceived walking ability (Table 4).

## Discussion

New measures to detect disability and its progression are claimed in MS for both clinical practice and trial design. In this cross-sectional study we investigated the applicability and feasibility of inertial sensor-based gait analysis in MS.

We first found that in both MS and HC subjects there was an excellent repeatability of most of the motor performance parameters evaluated in a single session. Then, a multivariable model revealed that two kinematic variables (E and mROM_H_) were able to independently distinguish MS from control group. The mROM_H_ measured motor impairment, being significantly correlated with walking speed and pyramidal functional score, whereas E was likely to assess overall disability, being correlated to EDSS even regardless motor strength. In line with this, E correlated very well with both clinically assessed and self-reported physical fatigue. Presently, there is no ‘‘gold standard” for detecting motor fatigue. Commonly, fatigue is assessed by several questionnaires including the FSS [19] and MFIS [18], based on patients’ self-assessments of the general condition. Hence, an objective tool for assessing motor fatigue in MS is crucial for a more precise diagnosis and for the design of treatment and rehabilitation programs. Recently, measurement of fatigue in term of decline in walking speed or change in gait pattern, have been proposed [14],[22]. Here, we have introduced a numerical index to objectively assess gait variability. E correctly classified patients with MS into fatigue and non-fatigue groups, better than validated questionnaires and even before the appearance of motor fatigue.

Furthermore, E index was able to discriminate progressive disease, indicating a new tool for monitoring disability among RRMS subjects. To date, no reliable markers of progressive disease course are available. Longitudinal studies will be useful to assess E changes during RR-SP transition phase and verify their prognostic value. On the other hand, mROM_H_ was also able to distinguish HC from MS patients with very low EDSS scores: a significant difference was already evident for patients with EDSS<2.0, demonstrating a high sensitivity in detecting a subclinical impairment. Further longitudinal studies will be useful to investigate the sensitivity of the mROM_H_ in early detecting disability progression.

In conclusion, we established two novel observer-independent measures of disability. E discriminated MS patients according to disability, whereas mROM_H_ was extremely sensitive in measuring motor impairment within patients. If confirmed in larger studies, this sensitivity will be of crucial importance for monitoring disease course and treatment effects in RRMS patients, when changes in the EDSS are small or absent, and in progressive MS patients, when also small and slow changes in the EDSS are the primary outcomes to assess.
